# Evaluation of Factors Associated With Appropriate Drug Prescription and Effectiveness of Informative and Educational Interventions—The EDU.RE.DRUG Project

**DOI:** 10.3389/fphar.2022.832169

**Published:** 2022-04-25

**Authors:** Federica Galimberti, Elena Olmastroni, Manuela Casula, Ivan Merlo, Matteo Franchi, Alberico Luigi Catapano, Valentina Orlando, Enrica Menditto, Elena Tragni, on behalf of EDU.RE.DRUG Group

**Affiliations:** ^1^ IRCCS MultiMedica, Sesto S. Giovanni, Italy; ^2^ Epidemiology and Preventive Pharmacology Service (SEFAP), Department of Pharmacological and Biomolecular Sciences, University of Milan, Milan, Italy; ^3^ National Centre for Healthcare Research and Pharmacoepidemiology, Milan, Italy; ^4^ Laboratory of Healthcare Research and Pharmacoepidemiology, Department of Statistics and Quantitative Methods, University of Milano-Bicocca, Milan, Italy; ^5^ CIRFF, Center of Pharmacoeconomics, Federico II University of Naples, Naples, Italy; ^6^ Department of Pharmacy, Federico II University of Naples, Naples, Italy

**Keywords:** pragmatic trial design, appropriate prescription, educational intervention, drug prescribing, feedback report

## Abstract

**Background:** EDU.RE.DRUG study is a prospective, multicentre, open-label, parallel-arm, controlled, pragmatic trial directed to general practitioners (GPs) and their patients.

**Methods:** The study data were retrieved from health-related administrative databases of four local health units (LHUs) of Lombardy and four LHUs in Campania. According to the LHUs, the GPs/patients were assigned to (A) intervention on both GPs (feedback reports about appropriate prescribing among their patients and online courses) and patients (flyers and posters on proper drug use), (B) intervention on GPs, (C) intervention on patients, and (D) no intervention (control arm). A set of appropriate prescribing indicators (potential drug–drug interactions [pDDIs], potential and unnecessary therapeutic duplicates [pTDs], and inappropriate prescriptions in the elderly [ERD-list]) were measured at baseline and after the intervention phase. The effectiveness of the intervention was evaluated estimating the absolute difference in percentages of selected indicators carrying out linear random-intercept mixed-effect models.

**Results:** A cohort of 3,586 GPs (2,567 in intervention groups and 1,019 in the control group) was evaluated. In Campania, the mean pre-intervention percentage of patients with at least one pDDI was always greater than 20% and always lower than 15% in Lombardy. The pre–post difference was quite heterogeneous among the LHUs, ranging from 1.9 to −1.4 percentage points. The mean pre-intervention percentage of patients with pTDs ranged from 0.59 to 2.1%, with slightly higher values characterizing Campania LHUs. The magnitude of the pre–post difference was very low, ranging from −0.11 to 0.20. In Campania, the mean pre-intervention percentage of patients with at least one ERD criterium was considerably higher than in Lombardy (approximately 30% in Lombardy and 50% in Campania). The pre–post difference was again quite heterogeneous. The results from the models accounting for GP geographical belonging suggested that none of the interventions resulted in a statistically significant effect, for all the three indicators considered.

**Conclusion:** The proposed strategy was shown to be not effective in influencing the voluntary changes in GP prescription performance. However, the use of a set of explicit indicators proved to be useful in quantifying the inappropriateness. Further efforts are needed to find more efficient strategies and design more tailored interventions.

## 1 Introduction

Since the general practitioner (GP)–patient interaction leads in most cases to a drug prescription, the prescribing quality in general practice is a crucial issue, having a significant impact on the well-being of patients and representing a substantial part of healthcare expenditure (Steinke et al., 1999; Mekonnen et al., 2021).

The failure to prescribe appropriate drug therapy, named “inappropriate prescribing,” occasionally simply results in the absence of any clinical effect. In other more serious cases, the consequences may lead to adverse events, namely, aggravation of the illness, additional diagnostic testing, and increased hospitalizations and mortality, especially in older people, or in comorbid individuals who may have compromised physiologic functions (Hamilton et al., 2009). Furthermore, the inappropriate use of medicines can lead to increasing costs for the patient and healthcare system and wastage of scarce health resources (Ofori-Asenso and Agyeman, 2016). Thus, inappropriate prescribing has become a global healthcare concern.

It is essential to identify potentially inappropriate prescribing (PIP) and correct and optimize it where necessary, with the expectation that this will avoid serious harm (Avery, 2008). A wide range of interventions can be implemented to change patients’ and prescribers’ behaviour to improve drug prescribing. Among them, educational/professional strategies are often aimed at persuading or informing, and this usually involves the use of printed materials, seminars, bulletins, and face-to-face interventions. Continuing medical education (CME) is the most common educational intervention, delivered through various methods, namely, interactive teaching complemented by a decision algorithm, mailed educational material combined with individualized feedback, and face-to-face visits to physicians (Kaur et al., 2009).

The EDU.RE.DRUG (effectiveness of informative and/or educational interventions aimed at improving the appropriate use of drugs designed for general practitioners and their patients) study has been designed to deeply investigate the practice of prescribing among general practitioners in two Italian regions, to highlight the most frequent events of inappropriateness, and to plan tailored intervention for GPs and patients focused on this critical issue. The primary objective was to assess the effectiveness of informative and/or educational interventions addressed to general practitioners and their patients, aimed at improving the prescribing quality and promoting proper medication use.

## 2 Methods

### 2.1 Study Design

EDU.RE.DRUG was a prospective, pragmatic, multicentre, open-label, parallel-arm, and controlled trial, started in April 2017, which has been extensively described elsewhere (Casula et al., 2020). Briefly, it was a non-randomized, open-label, cluster intervention design. All experimental units (GPs and/or patients) in each cluster receive the scheduled treatment. Eight Local Health Units (LHUs) were enrolled: four in the Campania region, in the southern part of Italy, and four in the Lombardy region, in the north of Italy.

### 2.2 Study Setting and Data Source

The clinical setting of the study was the general practice. The study population was composed of all GPs and all their adult patients aged ≥40 years from the eight LHUs involved.

Data were retrieved from health-related administrative databases containing demographic and pharmacy-refill data of all beneficiaries of the National Health Service (NHS) in the LHUs involved (*Bergamo*, *Lecco*, *Mantova*, and *Monza-Brianza* in Lombardy and *Avellino*, *Caserta*, *Napoli 1 Centro*, and *Napoli 2 Nord* in Campania):

Compliance with national and European laws on personal data was guaranteed by the LHUs through the generation of unique anonymous codes for each patient and each prescriber, with respect to the privacy of every citizen.

### 2.3 Definition of Inappropriate Prescribing Indicators

Prescription of potential drug–drug interactions (pDDIs) was defined based on MediRisk software (INXBASE https://ravimid.med24.ee/Accessed). In this project, two drugs were considered potentially interacting if their coverage periods (calculated since their dispensation date and based on their defined daily doses) overlapped for at least 1 day. Only pDDIs with major clinical significance (excluded those with low level of documentation) or contraindicated clinical significance (regardeless the level of documentation) were considered.

Potential and unnecessary therapeutic duplicates (pTDs) were defined as two or more prescribed drugs with the same ATC code at the second or third or fourth level but a different ATC code at the fifth level (Fulda et al., 2004) with at most 3 days between the two dispensation dates. Specifically, we selected the following classes:

• Drugs for peptic ulcer and gastro-oesophageal reflux disease (ATC codes: A02Bxxx and A02Bxxx).
• Agents acting on the renin–angiotensin system (ATC codes: C09Axxx/C09Bxxx and C09Cxxx/C09Dxxx).
• Statins (ATC codes: C10AAxx/C10BAxx and C10AAxx/C10BAxx).
• Antipsychotics (ATC codes: N05Axxx and N05Axxx).

Only in the elderly population (aged ≥65 years), we defined the ERD-list (EDU.RE.DRUG list; Casula et al., 2020) developed based on the updated Beers criteria (Radcliff et al., 2015), the STOPP&START criteria (O' Mahony et al., 2018), and the EU-(7)-PIM list (Renom-Guiteras et al., 2015). The three lists were merged and adapted to Italian settings by selecting only drugs available on the Italian market and reimbursed by Italian NHS. Moreover, the selection was limited to drugs always to be avoided in elderly patients, excluding drugs that should be used with caution or avoided in certain patients with certain diseases or conditions, as these circumstances cannot be evaluated through administrative databases.

### 2.4 Study Intervention

The GPs and their patients were assigned to one of the following arms: (A) intervention on GPs and patients; (B) intervention on GPs; (C) intervention on patients; and (D) control group. The intervention addressing GPs consisted in feedback reports (describing inappropriate prescription status of their patients in comparison to the median levels of their own LHUs) and a free online Continuing Medical Education (CME) course about rational prescribing and appropriateness measurement. Notably, participation to CME course was not mandatory.

The intervention designed for patients consisted in flyers and posters distributed in GPs’ ambulatories and community pharmacies, focusing on correct drug use (efficacy/safety, adherence to GP indications, and self-medication).

### 2.5 Study Outcome

The primary outcome was the changes in prevalence of inappropriate prescribing indicators among GPs, assessed through the pre-specified indicators, after the interventions vs. baseline.

Among the secondary outcomes (Casula et al., 2020), here we have reported results about the identification of predictors of poor prescription appropriateness. We firstly evaluated potential predictors in an attempt to identify major covariates to be accounted for in the assessment of the primary outcome.

### 2.6 Statistical Analysis

#### 2.6.1 Covariates

Several covariates have been assessed, both at the patient’s and GP’s levels.

From demographic databases, we retrieved birth date and sex of each patient. Using pharmacy-refill and hospitalization databases, we estimated the Charlson Comorbidity Index (Charlson et al., 1987), indicating the comorbidity status.

From demographic databases, we retrieved birth date, sex, and number of registered patients of each GP. We also calculated the percentage of elderly patients. Using pharmacy-refill databases, we estimated the annual number of different active drugs prescribed by each GP (drug portfolio).

#### 2.6.2 Identification of Determinants of Inappropriate Prescribing

Considering only the data relating to the period prior to intervention, analyses were conducted to evaluate the effect of several variables (related to patients, GPs, or LHUs) on the probability of being exposed to 1) pDDIs, 2) pTDs, and 3) inappropriate prescriptions according to the ERD-list. Each of the three outcomes was analysed using a set of four models that differed from each other in the number of covariates used. All the models were logistic random intercepts models complying with the hierarchy data structure in which patients (level 1) were nested within the GPs (level 2), nested within the LHUs (level 3). The dependent variable (i.e., the outcome) assumed value 1 if the patient had been exposed to the inappropriateness indicator under exam, while it assumed value 0 otherwise.

Below are the covariates (fixed effects) included in each of the four models:

• Model 1: no covariates included.
• Model 2: patient level covariates included.
• Model 3: patient and GP level covariates included.
• Model 4: patient, GP, and LHU level covariates included.

In this application, GP and LHU are clustering variables. With the aim of investigating the amount of heterogeneity of each outcome explained by these variables, the median odds ratios (MORs) were calculated for each of the four models (Merlo et al., 2006). Given one clustering variable, the MOR is defined as the median value of the odds ratio between the cluster at the highest risk and the cluster at the lowest risk when randomly picking out two clusters. Hence, it can be conceptualized as the increased risk that (in median) a subject would have when moving to another cluster with a higher risk. The MOR has always values greater than 1, where 1 represents no variation between clusters, and increases as high as the between-cluster variation.

#### 2.6.3 Intervention Effectiveness (Pre–post Analysis)

The primary and secondary outcomes were evaluated in a 12-month period before intervention (pre-intervention phase, April 2016–March 2017) and in a 12-month period after the intervention (post-intervention phase, April 2018–March 2019). The analysis was based on GP, as statistical unit. The difference (Δ pre − post) in the outcomes was estimated separately for each LHU.

The pre–post evaluation was performed with respect to the following three outcomes:

• Absolute difference in the percentage of patients (age ≥40 years) with pDDIs between the period preceding the intervention and the following period.
• Absolute difference in the percentage of patients (age ≥40 years) with pTDs between the period preceding the intervention and the following period.
• Absolute difference in the percentage of elderly patients (age ≥65 years) with inappropriate prescriptions (according to the ERD-list) between the period preceding the intervention and the following period.

In order to study the effect of the interventions on the outcome (pre–post difference) and to take into account the hierarchical structure of the data, a linear random intercept mixed-effect model was used. The fixed components were: GPs' characteristics (sex, age, number of patients, percentage of elderly patients, and drug portfolio), intervention arm, and pre-intervention percentage of the evaluated indicator. The random intercepts represented the administrative areas. The fit of the models was evaluated considering the marginal and conditional *R* ^2^ (Nakagawa and Schielzeth, 2013).

Finally, a stratified analysis was conducted in order to evaluate the effect of GP characteristics on the outcome dividing the physicians with respect to the region of belonging. In both strata, there were four administrative areas and four different interventions (one per area). Under this setting, it is no longer possible to distinguish the effect of the intervention from the effect of the area. However, the effect of the other variables remains interpretable.

#### 2.6.4 Data Analysis

Continuous variables are presented as means and standard deviations (SD) or medians and interquartile ranges (IQR), whereas categorical variables are presented as cases (N) and percentage rate (%). In all analyses, a *p*-value <0.05 was considered statistically significant. All analyses were performed using the SAS software (version 9.4; SAS Institute, Cary, NC, United States ).

### 2.7 Ethics

The study was approved by the Ethics Committee of the University of Milan on 07 June 2017 (code 15/17).

Procedures aimed at protecting personal data will be implemented in order to safeguard privacy and to prevent the identification of individual data (according to the Italian law D.Lgs. n. 196/2003). Anonymized regional administrative data can be used without a specific written informed consent when patient information is collected for healthcare management and healthcare quality evaluation and improvement (according to art. 110 on medical and biomedical and epidemiological research, Legislation Decree 101/2018).

## 3 Results

A cohort of 3,586 GPs was evaluated; the pre-intervention characteristics are listed in Table 1. The mean age of the GPs was quite similar among the administrative areas, while there was a greater presence of female GPs in the Lombardy areas. Physicians from *Napoli 2 Nord* LHU had an average percentage of elderly patients lower than that of the other LHUs.

**TABLE 1:** GPs pre-intervention characteristics in the eight Local Health Units. Sex is reported as frequency (%); other variables are considered on a continuous scale and are reported as mean ± SD.

	*Monza-Brianza* Lombardy	*Caserta* Campania	*Mantova* Lombardy	*Avellino* Campania	*Bergamo* Lombardy	*Napoli 1 Centro* Campania	*Lecco* Lombardy	*Napoli 2 Nord Campania*
	N = 477	N = 542	N = 243	N = 280	N = 591	N = 602	N = 196	N = 655
	None		Patients		GPs		GPs and patients	
Male sex, N (%)	290 (60.8)	410 (75.7)	174 (71.6)	210 (75.0)	392 (66.3)	470 (78.1)	138 (70.4)	533 (81.4)
Age (years), mean ± SD	58.6 ± 6.9	60.5 ± 4.2	59.1 ± 6.2	60.6 ± 4.2	57.1 ± 6.8	61.1 ± 4.0	57.7 ± 7.1	59.5 ± 4.4
Patients (N), mean ± SD	1,489.7 ± 191.5	1,246.1 ± 382.6	1,328.2 ± 313.2	1,187.2 ± 402.2	1,422.2 ± 284.0	1,208.7 ± 362.6	1,462.7 ± 212.5	1,205.8 ± 389.8
Elderly patients (%), mean ± SD	25.8 ± 5.6	19.8 ± 5.3	25.8 ± 6.0	24.1 ± 5.6	22.9 ± 5.6	22.9 ± 5.6	25.2 ± 5.2	14.8 ± 4.3
Drug portfolio* (N), mean ± SD	332.2 ± 29.6	348.8 ± 53.2	317.9 ± 31.1	343.1 ± 60.4	321.6 ± 34.1	368.2 ± 55.1	321.2 ± 29.1	345.9 ± 53.5

GP, general practitioner. *Drug portfolio: annual number of different active drugs prescribed by the GP.

### 3.1 Identification of Predictors of Inappropriate Prescribing

#### 3.1.1 Predictors of Exposure to Potential Drug–Drug Interactions

Results for the fully adjusted model, considering the exposition to pDDI as outcome, are reported in Table 2. The risk of being exposed to pDDIs increased with increasing age of the patient (for each 10-year increase: OR 1.53, 95% CI 1.52–1.53). Compared to patients with the Charlson Comorbidity Index equal to zero, patients with higher values showed higher risk; patients with values between 3 and 4 were the most likely to be exposed to interactions (OR 3.17, 95% CI 3.08–3.25). Patients from Campania (OR 1.24, 95% CI 1.10–1.39) had a higher risk, while male patients were less exposed to interactions (OR 0.74, 95% CI 0.73–0.75). The risk decreased with increase in the number of patients per GP (OR 0.95, 95% CI 0.95–0.96). The notable decrease in the MOR at the LHU level passing from Model 2 to Model 3 (from 1.40 to 1.13; data not shown) suggested that the differences in distribution of GP’s characteristics between LHUs played a key role in determining the inhomogeneity of the outcome among territories.

**TABLE 2:** Results for the fixed effects of the fully adjusted models.

Outcomes	Odds ratio (95% CI)		
	Potential drug–drug interactions	Potential therapeutic duplicates	Inappropriate prescriptions in the ERD-list
**Patient**			
Sex			
Female	Ref	Ref	Ref
Male	0.74 (0.73–0.75)	1.10 (1.07–1.13)	0.78 (0.78–0.79)
Age (10-year increase)	1.53 (1.52–1.53)	1.42 (1.41–1.43)	1.15 (1.14–1.15)
Charlson Comorbidity Index			
0	Ref	Ref	Ref
1-2	2.28 (2.26–2.31)	1.87 (1.81–1.94)	1.42 (1.40–1.44)
3-4	3.17 (3.08–3.25)	2.36 (2.22–2.50)	1.74 (1.68–1.79)
≥5	2.78 (2.69–2.88)	2.94 (2.74–3.17)	1.77 (1.70–1.84)
**Patient’s GP**			
Sex			
Female	Ref	Ref	Ref
Male	1.04 (1.02–1.07)	1.04 (0.98–1.11)	1.08 (1.04–1.11)
Age (10-year increase)	1.00 (0.99–1.02)	1.03 (0.99–1.08)	1.03 (1.01–1.05)
Number of patients (100-unit increase)	0.95 (0.95–0.96)	0.88 (0.87–0.89)	0.95 (0.95–0.96)
Percentage of elderly patients (1-percentage point increase)	0.97 (0.97–0.98)	0.96 (0.95–0.96)	0.97 (0.97–0.97)
Number of distinct drugs prescribed (10-unit increase)	1.07 (1.06–1.06)	1.14 (1.13–1.16)	1.07 (1.06–1.07)
**Patient’s LHU**			
Location			
Lombardy	Ref	Ref	Ref
Campania	1.24 (1.10–1.39)	0.83 (0.68–1.03)	1.48 (1.25–1.75)

GP, general practitioner; LHU, local health unit.

#### 3.1.2 Predictors of Exposure to Potential Therapeutic Duplicates

Results for fully adjusted model for pTD prescriptions (Table 2) show that male (OR 1.10, 95% CI 1.07–1.13) and older patients (for each 10-year increase: OR 1.42, 95% CI 1.41–1.43) had a higher risk of being exposed to duplicate drug prescriptions. Furthermore, the risk increased as the Charlson Comorbidity Index increases (for scores 3–4: OR 2.36, 95% CI 2.22–2.50; for scores ≥5: OR 2.94, 95% CI 2.74–3.17). GPs with a large number of patients were less likely to prescribe therapeutic duplicates (OR 0.88, 95% CI 0.87–0.89) while the risk increased for GPs with a higher number of distinct drugs prescribed (for each 10-unit increase: OR 1.14, 95% CI 1.13–1.16). The MOR at the GP level was quite large even in the fully adjusted model (1.60), showing that the frequency of the outcome was largely attributable to the physician and to his/her unobserved characteristics. The MOR at the LHU level decreased from 1.49 to 1.13 passing from Model 2 to Model 3.

#### 3.1.3 Predictors of Exposure to Inappropriate Drugs in the ERD-list

Results for the fully adjusted model (Table 2) show that the risk for a patient to be exposed to ERD drugs significantly increased with increasing age (for each 10-year increase: OR 1.15, 95% CI 1.14–1.15), Charlson Comorbidity Index (for scores 3–4: OR 1.74, 95% CI 1.68–1.79; for scores ≥5: OR 1.77, 95% CI 1.70–1.84). Moreover, the risk was higher in Campania (OR 1.48, 95% CI 1.25–1.75) and in patients registered with a male GP (OR 1.08, 95% CI 1.04–1.11). Conversely, the risk was lower in male patients (OR 0.78, 95% CI 0.78–0.79) and decreased with an increase in the number of patients per GP and in the percentage of elderly people assisted.

The MOR at the LHU level greatly decreased (from 1.58 to 1.24; data not shown) when adding GP characteristics in the model, showing that a part of the between-LHU variation in the outcome was attributable to the fact that in different LHUs, there were physicians with different characteristics.

### 3.2 Pre–Post Analysis

#### 3.2.1 Efficacy of Intervention on Potential Drug–Drug Interactions

The mean percentage (SD) of patients with pDDIs in the pre-intervention period and the mean value of the absolute difference between the pre- and post-intervention percentages for the eight areas are reported in Supplementary Table S1. In Campania, the mean pre-intervention percentage of pDDI patients was always greater than 20%, while it was always lower than 15% in Lombardy. The pre–post difference was quite heterogeneous between the areas, ranging from 1.9 to −1.4 percentage points.

The results from a linear random intercept model accounting for GPs geographical belonging are shown in Table 3. Before applying the model, the numerical covariates were centred with respect to the mean. Therefore, the intercept represents the average outcome (pre–post difference) for a female GP whose covariates were equal to the average values observed in the data set and underwent no intervention. None of the interventions resulted in a statistically significant effect compared to the group that did not receive any intervention. An increase in the age of the GP or the pre-intervention percentage of pDDI patients was significantly associated with a decrease in the percentage of patients with pDDI from the pre-intervention period to the subsequent. Conversely, higher percentage of elderly patients or higher number of distinct drugs prescribed in the pre-intervention period was significantly associated with lower pre–post difference.

**TABLE 3:** Linear random intercept model on pDDIs, pTDs, and ERD prescription.

Outcomes	Potential drug–drug interactions			Potential therapeutic duplicates			Inappropriate prescriptions in the ERD-list		
**Fixed effects**									
**Parameter**	**Estimate (95% CI)**		***p*-value**	**Estimate (95% CI)**		***p*-value**	**Estimate (95% CI)**		***p*-value**
Intercept*	0.17 (−6.19; 6.52)		0.945	0.13 (−0.07; 0.32)		0.148	3.62 (−3.90; 11.13)		0.252
Intervention									
None	Ref		-	Ref		-	Ref		-
GPs	1.19 (−7.78; 10.17)		0.731	0.04 (−0.21; 0.30)		0.652	0.76 (−9.83; 11.34)		0.852
Patients	0.93 (−8.05; 9.92)		0.787	0.13 (−0.14; 0.40)		0.243	2.65 (−7.97; 13.26)		0.527
GPs and patients	1.15 (−7.83; 10.13)		0.741	−0.06 (−0.32; 0.20)		0.560	2.48 (−8.12; 13.09)		0.551
GP sex									
Female	Ref		-	Ref		-	Ref		-
Male	−0.27 (−0.68; 0.13)		0.155	−0.10 (−0.20; −0.01)		0.037	−1.23 (−2.05; −0.42)		0.009
GP age (1-year increase)	0.06 (0.03; 0.09)		<0.001	0.08 (0.02; 0.15)		0.012	0.23 (0.17; 0.28)		<0.001
Number of patients (100-unit increase)	−0.06 (−0.14; 0.03)		0.189	0.06 (0.04; 0.08)		<0.001	0.17 (0.003; 0.33)		0.046
Percentage of elderly patients (1-percentage point increase)	−0.03 (−0.06; −0.001)		0.042	0.08 (0.02; 0.15)		0.014	0.07 (0.002; 0.14)		0.042
Percentage of pre-intervention patients with relevant inappropriateness (1-percentage point increase)	0.40 (0.37; 0.42)		<0.001	0.37 (0.33; 0.40)		<0.001	0.38 (0.35; 0.41)		<0.001
Number of distinct drugs prescribed (10-unit increase)	-0.15 (-0.21; -0.08)		<0.001	-0.05 (-0.06; -0.03)		<0.001	-0.33 (-0.46; -0.20)		<0.001
**Random effects**									
**Parameter**	**Estimate**	***p*-value**	**ICC**	**Estimate**	***p*-value**	**ICC**	**Estimate**	***p*-value**	**ICC**
Random intercept variance	10.41	0.081	<0.36	0.01	0.284	0.01	14.38	0.09	0.16
Residual variance	18.73	0.001		1.04	<0.001		75.69	<0.001	
**Model fit**									
Marginal R^2^	0.28				0.13			0.32	
Conditional R^2^	0.54				0.14			0.43	

GP, general practitioner. ICC, intra-class correlation coefficient. *Numerical covariates were centred with respect to the mean. Therefore, the intercept represents the average outcome (pre-post difference) for a female GP whose covariates were equal to the average values observed in the dataset and underwent no intervention.

Stratifying by region (Supplementary Table S2), a higher percentage of elderly patients was significantly associated with lower pre–post difference in the Lombardy LHUs, while the association was not significant in Campania LHUs. Globally, no relevant differences were found between the two regions.

#### 3.2.2 Efficacy of Intervention on Potential Therapeutic Duplicates

The mean pre-intervention percentage of patients with duplicate drugs (Supplementary Table S3) was low in all the LHUs, ranging from 0.59 to 2.1%; slightly higher values characterized the Campania LHUs. The magnitude of the pre–post difference was very low, ranging from −0.11 to 0.20.

The results from a linear random intercept model accounting for GPs geographical belongings are shown in Table 3. None of the interventions resulted in a statistically significant effect compared to the control group. The increase in the age of the GP, number of patients, percentage of elderly patients, and pre-intervention percentage of patients with duplicate drugs was significantly associated with a decrease in the percentage of patients with duplicate drugs from the pre-intervention period to the subsequent. Conversely, male sex and an increased number of distinct drugs prescribed in the pre-intervention period were significantly associated with lower pre–post difference.

The age of the GP was directly associated with pre–post difference in Campania, while the association was not significant for the GPs from Lombardy. Conversely, the percentage of elderly patients was directly associated with pre–post difference in Lombardy LHUs, while no significant association was found for Campania LHUs (Supplementary Table S4).

#### 3.2.3 Efficacy of Intervention on Prescription of Drug in the ERD-list

In Campania, the mean pre-intervention percentage of patients exposed to ERD drugs was considerably higher than in Lombardy (Supplementary Table S5). The pre–post difference was quite heterogeneous between the areas. On average, *Napoli 2 Nord* LHU showed the greatest reduction in the percentage of patients with inappropriate prescriptions (+11.3 percentage points), while GPs from *Lecco* LHU increased the percentage of patients with prescriptions of drug in the ERD-list on average by 0.5 percentage points.

The effect of the intervention arm and GP characteristics on the pre–post difference in the percentage of patients exposed to ERD drugs is shown in Table 3. None of the interventions resulted in a statistically significant effect compared to the group that did not receive any intervention. The increases in the age of the GP, number of patients, percentage of elderly patients, and pre-intervention percentage of patients with inappropriate prescriptions (ERD-list) were significantly associated with a decrease in the percentage of patients with ERD drugs in the post-intervention period, compared to the pre-intervention period. Conversely, male sex and a higher number of distinct drugs prescribed in the pre-intervention period were significantly associated with lower pre–post difference.

The results of the analysis stratified by region (Supplementary Table S6) showed that an increase in the age of the GP resulted in greater pre–post difference in both regions, with the effect that appears to be more pronounced in Campania (0.49 vs. 0.08). An increase in the number of drugs prescribed by the GP in the pre-intervention period was significantly associated with a lower value of the outcome in Campania, while no association was found for the areas of Lombardy.

## 4 Discussion

Medication prescription is one of the most powerful tools for GPs in the prevention and treatment of diseases and the alleviation of symptoms. However, drug-related problems represent an important source of patient morbidity, and many cases of which could be prevented through the highest-quality medicine prescribing and management (Howard et al., 2003; Pirmohamed et al., 2004; Howard et al., 2007; Howard et al., 2008).

Our first analysis allowed to investigate whether some factors could affect the prescriptive inappropriateness or not. The results for fixed and random effects models show that the risk for a patient to be exposed to ERD drugs, pDDIs, or pTDs significantly increased with increasing age and Charlson Comorbidity Index. Male sex decreased the risk of being prescribed with ERD drugs or pDDIs, while increased the risk of pTDs. Regarding GPs' characteristics, the risk was higher with male sex, increased with the number of distinct drugs prescribed by the physician, and decreased with the increase in the number of patients per GP and in the percentage of elderly people assisted. The age of the GPs seemed not to have a major influence on the probability of inappropriate prescriptions, even if we observed a trend (statistically significant only for ERD drug prescriptions) towards an increased risk with increasing GP age. Consistent with literature, our data confirmed that the presence of comorbidities and the concomitant use of multiple drugs increase the risk of inappropriateness. In a German study (Stock et al., 2014), the risk to receive an inappropriate prescription in an elderly cohort increased with age, and women had a significantly higher risk compared with men. Previous studies from the United States and other German cohorts consistently reported age and female sex as risk factors for receiving inappropriate prescriptions. Moreover, comorbidity was identified as an additional risk factor for potentially inappropriate medication prescription (Almeida et al., 2019; Magalhaes et al., 2020). Regarding GPs characteristics, some studies also showed that female prescribers may be more likely to prescribe carefully and conservatively than male prescribers; evidence suggests that female physicians spend more time with their patients and are more likely to adhere to guidelines (Rochon et al., 2018; Mishra et al., 2020). Finally, the geographical variability in prescribing performance was observed also in other studies. This could be explained by variations in the health condition of the population, socioeconomic differences, and also the independent local management of the GPs by the LHUs (Lund et al., 2013; Saastamoinen and Verho, 2021).

A wide range of interventions can be implemented to change patients’ and prescribers’ behaviour and ameliorate drug prescribing and use. This could lead to significant improvements in patient outcomes and effective use of healthcare expenditure. Within the EDU.RE.DRUG project, an audit and feedback plus educational interventions approach was delivered in a prospective, pragmatic, multifactorial, open-label, parallel-armed, controlled trial. In the evaluation of the effectiveness of our intervention, the results from a linear random intercept model accounting for GPs geographical belonging showed that none of the interventions resulted in a statistically significant effect compared to the control group (the intervention did not effectively influence the voluntary changes in prescription performance by GPs).

Trying to examine potential explanation, we first considered the role of LHU heterogeneity. As in our study, the single statistical units (GPs) were not randomly assigned to the interventions, but the assignment of interventions took place by clusters (administrative areas), and to isolate the effect of the interventions from the effect of the groups (if a group effect is present) is challenging. The power of the study in identifying the effect of a treatment is influenced not only by the factors common to a classical randomized and controlled study but also by the intra-class correlation coefficient (ICC) and the number of groups assigned to each intervention. To address this point, a simulation analysis was carried out. Different scenarios were hypothesized (i.e., different combination of ICC and intervention effect values), and 2,000 data sets were simulated for each scenario. Each data set was analysed using a mixed effects model, as done in the main analysis. The results from these analyses showed that the difficulty in detecting even small variations in the outcomes of interest is partly attributable to the heterogeneity of the LHU involved, an aspect necessarily present in the setting of a pragmatic trial.

Other interventions aimed at improving GP’s prescribing practice, conducted using pragmatic trials, have often not been successful. In a pragmatic, cluster randomized controlled trial performed in Germany (Muth et al., 2018), participants (≥60 years, ≥3 chronic conditions under pharmacological treatment, and ≥5 long-term drug prescriptions with systemic effects) were involved in a checklist-based interview on medication-related problems. Assisted by a computerised decision support system, the GPs optimised medication, discussed it with their patients, and adjusted it accordingly. The control group was managed with the usual care. The primary outcome was a modified Medication Appropriateness Index (MAI) assessed in blinded medication reviews and calculated as the difference between the baseline and after 6 months. Intervention had no significant effect on the primary outcome. Another pre–post study in a tertiary Malaysian hospital aiming at investigating the impact of a multifaceted intervention with a smartphone app on physicians’ and clinical pharmacists’ behaviour (Akkawi et al., 2020) did not significantly affect the prevalence of potentially inappropriate medication among hospitalized older adults. A systematic review that aimed to determine which interventions, alone or in combination, would be effective in improving the appropriate use of polypharmacy and reducing medication-related problems in older people found no clear evidence of a clinically significant improvement for any specific type of intervention (Rankin et al., 2018).

The difficulty in demonstrating the effectiveness of interventions in pragmatic studies is partly due to the design itself. Indeed, in this type of trial, it is not possible to control the real application of the intervention as it would happen in a controlled explanatory study. In the EDU.RE.DRUG project, while potentially involving all the GPs and/or patients, no obligation to read and use the materials of the interventions was imposed. Therefore, it was not possible to force the active participation to the proposed activities (reports and CME course), nor the translation in a behavioural change. It was not even possible to check whether the GPs had read and/or taken into consideration the reports. Thus, the results of the project accounted also for the poor participation rate of the GPs involved.

Another possible explanation of our results can be found in the time windows used. If the intervention had an immediate effect, even minimal, possibly not maintained later on, it would have been evaluated immediately after its delivery. Unfortunately, we cannot know when (and if) the GP read the report or when he attended the CME course (made available for several months, in order to allow wider access). Therefore, the measurement of outcomes over a 1-year post-intervention period (for comparison with 1-year pre-intervention period) may have diluted the results. It could be useful to repeat the intervention, perhaps after 6 months to keep the GP’s attention on these issues.

Moreover, in the present study, the topics involved were many and wide-ranging, though all related to the problem of prescribing inappropriateness. We can argue that, behind the difficulty in modifying a consolidated and routine exercise, such as the prescription practice, this probably mitigates the strength of the intervention on several fronts, “confusing” and overloading the GP with respect to the inappropriateness to be changed and improved and the targets to be achieved.

### 4.1 Strengths and Limitations

We conducted a pragmatic trial, which may have limited the effect of the intervention, as the GPs were not obliged to consider the proposed material and to change their practice. Nevertheless, the study design has the enormous advantage of being very close to real clinical practice and is essential to improve prescription appropriateness in a real-life context.

In our study, we used secondary data, as they are routinely gathered at the individual level for administrative purposes and as a part of the healthcare system in Italy. The use of the existing data represents a powerful and relatively low-cost research tool; however, drugs traced in these databases are limited to those that are reimbursed by the Italian NHS (class A drugs), probably leading to an underestimation of PIP prevalence. In addition, these administrative databases do not contain information on the patient clinical history (together with other lifestyle and sociodemographic factors that could drive the choice of drug prescriptions), GP instructions, dose and times of administration, or indication for treatment. Therefore, the rationale for prescribing or starting medications is not known and patients might be wrongfully classified as being prescribed an inappropriate drug. Despite these limitations, large population administrative databases would have several advantages, such as the detection of different patterns of prescribing in the real world setting and the analysis of the complexity of drug prescriptions. They are a great source of information on drug utilization and GPs’ behaviours in routine clinical practice.

### 4.2 Implications for Practice

The evidence obtained from this project certainly has a relevant clinical value and can inform decision-making strategies and future research insights.

*Prescription inappropriateness is a relevant problem in our territory, with greater burden in elderly population.* Although there was no significant improvement overall, this study allowed to identify which are the most critical areas where strategies can be implemented to support the improvement of medication appropriateness. In the national context, the difference between regions is also an important alarm bell, in the perspective of making access and management homogeneous. These results can be a starting point for further studies focusing only on specific situations.

*Medical practice is difficult to change and more effective strategies must be found.* Evidence has shown that the acceptance of recommendations by GPs plays a critical role in the achievement of results, but there is no consensus on which is the best strategy. Future studies should ensure greater methodological rigor in the evaluation of interventions to reduce the prevalence of inappropriate prescriptions. Further evaluations are required to investigate the effectiveness of other types of individual and combined interventions. Qualitative studies involving health professionals and patients can provide important information about barriers for the implementation or acceptance of an intervention. As already discussed, it would be interesting to evaluate the effects of more focused and repeated interventions over time. We can also note that the tools implemented (reports and CME course) are routinely used by the LHUs for educational/training purposes and to direct the prescribing practice. It is therefore possible that, also given the nonmandatory nature of the intervention, these resources have been scarcely taken into consideration by doctors already highly exposed to these kinds of inputs. This may also suggest a general lack of effectiveness of these strategies usually used by local health authorities. It would therefore be advisable to find different intervention strategies, which can further stimulate the prescribers.

## 5 Conclusion

In our multifactorial pragmatic and controlled trial, we did not appreciate a decrease in potentially inappropriate prescriptions after 1-year follow-up. Implementing prescription practice with audit and feedback approaches was found to be poorly effective, overall, in primary care (Soleymani et al., 2019; Kroon et al., 2021). Nevertheless, our study allowed to identify factors associated with inappropriate prescribing, informing healthcare administrators and policy makers to better design corrective interventions.

Considering the limitations unveiled by our study, other strategies and management models should be designed, applied, and tested in order to lead to a relevant improvement in the overall prescribing qualities in the adult population.

## Data Availability

The data analyzed in this study is subject to the following licenses/restrictions: raw data were generated at the local health units. Derived data supporting the findings of this study are available from the corresponding author (MC) on request. Requests to access these data sets should be directed to Manuela Casula, manuela.casula@unimi.it.

## EDU.RE.DRUG Group

**Alberico L. Catapano**, Epidemiology and Preventive Pharmacology Service (SEFAP), Department of Pharmacological and Biomolecular Sciences, University of Milan, Milan, Italy; IRCCS MultiMedica, Sesto S. Giovanni, Milan, Italy; **Elena Tragni**, Epidemiology and Preventive Pharmacology Service (SEFAP), Department of Pharmacological and Biomolecular Sciences, University of Milan, Milan, Italy; **Enrica Menditto**, CIRFF, Center of Pharmacoeconomics, Department of Pharmacy, Federico II University of Naples, Naples, Italy; **Giovanni Corrao**, Department of Statistics and Quantitative Methods (DiSMeQ), University of Milano‐Bicocca, Milan, Italy; **Manuela Casula**, Department of Pharmacological and Biomolecular Sciences, University of Milan, Milan, Italy; IRCCS MultiMedica, Sesto S. Giovanni, Milan, Italy; **Federica Galimberti**, IRCCS MultiMedica, Sesto S. Giovanni, Milan, Italy; **Elena Olmastroni**, Epidemiology and Preventive Pharmacology Service (SEFAP), Department of Pharmacological and Biomolecular Sciences, University of Milan, Milan, Italy; **Veronica Russo**, CIRFF, Center of Pharmacoeconomics, Department of Pharmacy, Federico II University of Naples, Naples, Italy; **Ilaria Guarino**, CIRFF, Center of Pharmacoeconomics, Department of Pharmacy, Federico II University of Naples, Naples, Italy; **Sara Mucherino**, CIRFF, Center of Pharmacoeconomics, Department of Pharmacy, Federico II University of Naples, Naples, Italy **Valentina Orlando**, CIRFF, Center of Pharmacoeconomics, Department of Pharmacy, Federico II University of Naples, Naples, Italy; **Matteo Franchi**, Department of Statistics and Quantitative Methods (DiSMeQ), University of Milano‐Bicocca, Milan, Italy; **Ivan Merlo**, Department of Statistics and Quantitative Methods (DiSMeQ), University of Milano‐Bicocca, Milan, Italy; **Antonella Zambon**, Department of Statistics and Quantitative Methods (DiSMeQ), University of Milano‐Bicocca, Milan, Italy; **Arrigo Paciello**, Bergamo Local Health Authority, Bergamo, Italy; **Marco Gambera**, Bergamo Local Health Authority, Bergamo, Italy; **Rossana Piccinelli**, Bergamo Local Health Authority, Bergamo, Italy; **Samanta Sonzogni**, Bergamo Local Health Authority, Bergamo, Italy; **Valter Valsecchi**, Brianza (Lecco and Monza‐Brianza districts) Local Health Authority, Lecco, Italy; **Eugenio Scopinaro**, Brianza (Lecco and Monza‐Brianza districts) Local Health Authority, Lecco, Italy; **Sandro Rainer**, Valpadana (Mantova district) Local Health Authority, Mantova, Italy; **Alessia Speziali**, Valpadana (Mantova district) Local Health Authority, Mantova, Italy; **Simona Creazzola**, Napoli Centro Local Health Authority, Naples, Italy; **Michele Tari**, Caserta Local Health Authority, Caserta, Italy; **Mariano Fusco**, Napoli Nord Local Health Authority, Frattamaggiore (NA), Italy; **Ivana Lieto**, Avellino Local Health Authority, Avellino, Italy.

## References

[B1] Akkawi M. E. Nik Mohamed M. H. Md Aris M. A. (2020). The Impact of a Multifaceted Intervention to Reduce Potentially Inappropriate Prescribing Among Discharged Older Adults: a Before-And-After Study. J. Pharm. Pol. Pract 13, 39. 10.1186/s40545-020-00236-0 PMC7367269 32695426

[B2] Almeida T. A. Reis E. A. Pinto I. V. L. Ceccato M. D. G. B. Silveira M. R. Lima M. G. (2019). Factors Associated with the Use of Potentially Inappropriate Medications by Older Adults in Primary Health Care: An Analysis Comparing AGS Beers, EU(7)-PIM List , and Brazilian Consensus PIM Criteria. Res. Soc. Adm Pharm 15 (4), 370–377. 10.1016/j.sapharm.2018.06.002 29934277

[B3] Avery A. J. (2008). Prescribing Errors by Family Practice Residents. Postgrad. Med. J. 84 (990), 170–171. 10.1136/pgmj.2008.067892 18424571

[B4] Casula M. Menditto E. Galimberti F. Russo V. Olmastroni E. Scotti L. (2020). A Pragmatic Controlled Trial to Improve the Appropriate Prescription of Drugs in Adult Outpatients: Design and Rationale of the EDU.RE.DRUG Study. Prim. Health Care Res. Dev. 21 (e23), 1–7. 10.1017/S1463423620000249

[B5] Charlson M. E. Pompei P. Ales K. L. MacKenzie C. R. (1987). A New Method of Classifying Prognostic Comorbidity in Longitudinal Studies: Development and Validation. J. Chronic Dis. 40 (5), 373–383. 10.1016/0021-9681(87)90171-8 3558716

[B6] Fulda T. R. Lyles A. Pugh M. C. Christensen D. B. (2004). Current Status of Prospective Drug Utilization Review. J. Manag. Care Pharm. 10 (5), 433–441. 10.18553/jmcp.2004.10.5.433 15369426 PMC10437730

[B7] Hamilton H. J. Gallagher P. F. O'Mahony D. (2009). Inappropriate Prescribing and Adverse Drug Events in Older People. BMC Geriatr. 9, 5. 10.1186/1471-2318-9-5 19175914 PMC2642820

[B8] Howard R. Avery A. Bissell P. (2008). Causes of Preventable Drug-Related Hospital Admissions: a Qualitative Study. Qual. Saf. Health Care 17 (2), 109–116. 10.1136/qshc.2007.022681 18385404

[B9] Howard R. L. Avery A. J. Howard P. D. Partridge M. (2003). Investigation into the Reasons for Preventable Drug Related Admissions to a Medical Admissions Unit: Observational Study. Qual. Saf. Health Care 12 (4)**,** 280–285. 10.1136/Qhc.12.4.280 12897361 PMC1743731

[B10] Howard R. L. Avery A. J. Slavenburg S. Royal S. Pipe G. Lucassen P. (2007). Which Drugs Cause Preventable Admissions to Hospital? A Systematic Review. Br. J. Clin. Pharmacol. 63 (2), 136–147. 10.1111/j.1365-2125.2006.02698.x 16803468 PMC2000562

[B11] Kaur S. Mitchell G. Vitetta L. Roberts M. S. (2009). Interventions that Can Reduce Inappropriate Prescribing in the Elderly: a Systematic Review. Drugs Aging 26 (12)**,** 1013–1028. 10.2165/11318890-000000000-00000 19929029

[B12] Kroon D. Steutel N. F. Vermeulen H. Tabbers M. M. Benninga M. A. Langendam M. W. (2021). Effectiveness of Interventions Aiming to Reduce Inappropriate Drug Prescribing: an Overview of Interventions. J. Pharm. Health Serv. Res. 12 (3), 423–433. 10.1093/jphsr/rmab038

[B13] Lund B. C. Charlton M. E. Steinman M. A. Kaboli P. J. (2013). Regional Differences in Prescribing Quality Among Elder Veterans and the Impact of Rural Residence. J. Rural Health 29 (2), 172–179. 10.1111/j.1748-0361.2012.00428.x 23551647 PMC3940354

[B14] Magalhães M. S. Santos F. S. D. Reis A. M. M. (2020). Factors Associated with the Use of Potentially Inappropriate Medication by Elderly Patients Prescribed at Hospital Discharge. Einstein (Sao Paulo) 18, eAO4877. 10.31744/einstein_journal/2020AO4877 PMC6896600 31664332

[B15] Mahony D. O. Sullivan D. O. Byrne S. Connor M. N. O. Ryan C. Gallagher P. (2018). Corrigendum: STOPP/START Criteria for Potentially Inappropriate Prescribing in Older People: Version 2. Age Ageing 47 (3), 489. 10.1093/ageing/afx178 PMC5920325 29182733

[B16] Mekonnen A. B. Redley B. de Courten B. Manias E. (2021). Potentially Inappropriate Prescribing and its Associations with Health-Related and System-Related Outcomes in Hospitalised Older Adults: A Systematic Review and Meta-Analysis. Br. J. Clin. Pharmacol. 87 (11), 4150–4172. 10.1111/bcp.14870 34008195 PMC8597090

[B17] Merlo J. Chaix B. Ohlsson H. Beckman A. Johnell K. Hjerpe P. (2006). A Brief Conceptual Tutorial of Multilevel Analysis in Social Epidemiology: Using Measures of Clustering in Multilevel Logistic Regression to Investigate Contextual Phenomena. J. Epidemiol. Community Health 60 (4), 290–297. 10.1136/jech.2004.029454 16537344 PMC2566165

[B18] Mishra A. Read S. H. Rochon P. A. (2020). Influence of Physician Sex and Gender on Prescribing Practices Among Older Adults. J. Am. Geriatr. Soc. 68 (12), 2764–2767. 10.1111/jgs.16851 33047303

[B19] Muth C. Uhlmann L. Haefeli W. E. Rochon J. van den Akker M. Perera R. (2018). Effectiveness of a Complex Intervention on Prioritising Multimedication in Multimorbidity (PRIMUM) in Primary Care: Results of a Pragmatic Cluster Randomised Controlled Trial. Bmj Open 8 (2), e017740. 10.1136/bmjopen-2017-017740 PMC5855483 29478012

[B20] Nakagawa S. Schielzeth H. (2013). A General and Simple Method for obtainingR2from Generalized Linear Mixed-Effects Models. Methods Ecol. Evol. 4 (2), 133–142. 10.1111/j.2041-210x.2012.00261.x

[B21] Ofori-Asenso R. Agyeman A. A. (2016). Irrational Use of Medicines-A Summary of Key Concepts. Pharmacy (Basel) 4 (4), 35. 10.3390/Pharmacy4040035 PMC5419375 28970408

[B22] Pirmohamed M. James S. Meakin S. Green C. Scott A. K. Walley T. J. (2004). Adverse Drug Reactions as Cause of Admission to Hospital: Prospective Analysis of 18 820 Patients. BMJ 329 (7456)**,** 15–19. 10.1136/bmj.329.7456.15 15231615 PMC443443

[B23] Radcliff S. Yue J. R. Rocco G. Aiello S. E. Ickowicz E. Hurd Z. (2015). American Geriatrics Society 2015 Updated Beers Criteria for Potentially Inappropriate Medication Use in Older Adults. J. Am. Geriatr. Soc. 63 (11), 2227–2246. 10.1111/jgs.13702 26446832

[B24] Rankin A. Cadogan C. A. Patterson S. M. Kerse N. Cardwell C. R. Bradley M. C. (2018). Interventions to Improve the Appropriate Use of Polypharmacy for Older People. Cochrane Database Syst. Rev. 9 (9), CD008165. 10.1002/14651858.Cd008165.Pub4 30175841 PMC6513645

[B25] Renom-Guiteras A. Meyer G. Thürmann P. A. (2015). The EU(7)-PIM List: a List of Potentially Inappropriate Medications for Older People Consented by Experts from Seven European Countries. Eur. J. Clin. Pharmacol. 71 (7), 861–875. 10.1007/s00228-015-1860-9 25967540 PMC4464049

[B26] Rochon P. A. Gruneir A. Bell C. M. Savage R. Gill S. S. Wu W. (2018). Comparison of Prescribing Practices for Older Adults Treated by Female versus Male Physicians: A Retrospective Cohort Study. PLoS One 13 (10), e0205524. 10.1371/journal.pone.0205524 30346974 PMC6197851

[B27] Saastamoinen L. Verho J. (2021). Regional Variation in Potentially Inappropriate Medicine Use in Older Adults. - A National Register-Based Cross-Sectional Study on Economic, Health System-Related and Patient-Related Characteristics. Res. Soc. Adm Pharm 17 (6), 1223–1227. 10.1016/j.sapharm.2020.08.018 33071213

[B28] Soleymani F. Rashidian A. Hosseini M. Dinarvand R. Kebriaeezade A. Abdollahi M. (2019). Effectiveness of Audit and Feedback in Addressing over Prescribing of Antibiotics and Injectable Medicines in a Middle-Income Country: an RCT. Daru 27 (1), 101–109. 10.1007/s40199-019-00248-5 30788839 PMC6593028

[B29] Steinke D. T. MacDonald T. M. Davey P. G. (1999). The Doctor-Patient Relationship and Prescribing Patterns. A View from Primary Care. Pharmacoeconomics 16 (6), 599–603. 10.2165/00019053-199916060-00001 10724789

[B30] Stock S. Redaelli M. Simic D. Siegel M. Henschel F. (2014). Risk Factors for the Prescription of Potentially Inappropriate Medication (PIM) in the Elderly : an Analysis of Sickness Fund Routine Claims Data from Germany. Wien Klin Wochenschr 126 (19-20), 604–612. 10.1007/s00508-014-0589-2 25216754

