# Supplementary material for: Evaluation of Factors Associated With Appropriate Drug Prescription and Effectiveness of Informative and Educational Interventions—The EDU.RE.DRUG Project
Source: Front Pharmacol. 2022 Apr 25;13:832169. doi: 10.3389/fphar.2022.832169 (PMC9081494; doi:10.3389/fphar.2022.832169)
Supplement: Supplementary file 1 [file DataSheet1.docx]

**Title:**

**Evaluation of factors associated with appropriate drug prescription and effectiveness of informative and/or educational interventions – The EDU.RE.DRUG Project**

**SUPPLEMENTARY MATERIAL**

**Supplementary Table 1**. Percentage of patients with pDDIs in the pre-intervention period and observed pre-post difference. Mean (SD).

| **Local Health Unit** | *Monza-Brianza*  **Lombardy** | *Caserta*  **Campania** | *Mantova*  **Lombardy** | *Avellino*  **Campania** | *Bergamo*  **Lombardy** | *Napoli 1 Centro*  **Campania** | *Lecco*  **Lombardy** | *Napoli 2 Nord*  **Campania** |
| --- | --- | --- | --- | --- | --- | --- | --- | --- |
| **Intervention** | **None** | | **Patients** | | **GPs** | | **GPs and Patients** | |
| **Percentage of pDDI patients (pre-intervention)** | 12.2 (3.1) | 23.9 (6.1) | 14.1 (6.4) | 22.4 (6.0) | 13.2 (3.2) | 25.0 (7.8) | 12.2 (3.1) | 26.1 (6.5) |
| **Pre-post difference in the percentage of pDDI patients** | -0.1 (1.9) | -1.4 (2.7) | 1.1 (5.4) | -0.3 (4.7) | 0.3 (2.0) | 1.3 (8.2) | 0.1 (2.4) | 1.9 (5.8) |

**Supplementary Table 2**. Linear model on pDDIs stratified by region

|  | **Estimate (95% CI)** | **p value** |
| --- | --- | --- |
| **LOMBARDY** |  |  |
| Intercept* | 0.45 (0.23; 0.67) | <0.001 |
| Intervention (Territory) |  |  |
| None (*Monza-Brianza*) | Ref. | - |
| GPs (*Bergamo*) | -0.48 (-0.72; 0.24) | <0.001 |
| Patients (*Mantova*) | 0.03 (-0.27; 0.34) | 0.829 |
| GPs and patients (*Lecco*) | 0.11 (-0.21; 0.43) | 0.492 |
| GP sex |  |  |
| Female | Ref. | - |
| Male | -0.02 (-0.23; 0.20) | 0.871 |
| GP age (1-year increase) | 0.02 (0.01; 0.04) | 0.005 |
| Number of patients (100-unit increase) | -0.02 (-0.08; 0.05) | 0.602 |
| Percentage of elderly patients (1-percentage point increase) | -0.11 (-0.13; -0.09) | <0.001 |
| Percentage of pre-intervention pDDI patients (1-percentage point increase) | 0.58 (0.55; 0.61) | <0.001 |
| Number of distinct drugs prescribed (10-unit increase) | -0.06 (-0.12; -0.003) | 0.040 |
| **CAMPANIA** |  |  |
| Intercept* | -0.88 (-1.51; -0.25) | 0.007 |
| Intervention (Territory) |  |  |
| None (*Caserta*) | Ref. | - |
| GPs (*Napoli 1 Centro*) | 2.51 (1.86; 3.17) | <0.001 |
| Patients (*Avellino*) | 1.52 (0.71; 2.32) | <0.001 |
| GPs and patients (*Napoli 2 Nord*) | 2.42 (1.75; 3.09) | <0.001 |
| GP sex |  |  |
| Female | Ref. | - |
| Male | -0.32 (-0.90; 0.25) | 0.272 |
| GP age (1-year increase) | 0.13 (0.07; 0.19) | <0.001 |
| Number of patients (100-unit increase) | -0.05 (-0.17; 0.0.08) | 0.454 |
| Percentage of elderly patients (1-percentage point increase) | -0.01 (-0.07; 0.04) | 0.600 |
| Percentage of pre-intervention pDDI patients (1-percentage point increase) | 0.36 (0.32; 0.40) | <0.001 |
| Number of distinct drugs prescribed (10-unit increase) | -0.18 (-0.28; -0.08) | <0.001 |

GP general practitioner

* Within each region, numerical covariates were centred with respect to the mean. Therefore, the intercept represents the average outcome (pre-post difference) in the corresponding region for a female GP whose covariates were equal to the average values observed in the dataset and undergo no intervention.

**Supplementary Table 3**. Percentage of patients with pTDs in the pre-intervention period and observed pre-post difference. Mean (SD).

| **Local Health Unit** | *Monza-Brianza*  **Lombardy** | *Caserta*  **Campania** | *Mantova*  **Lombardy** | *Avellino*  **Campania** | *Bergamo*  **Lombardy** | *Napoli 1 Centro*  **Campania** | *Lecco*  **Lombardy** | *Napoli 2 Nord*  **Campania** |
| --- | --- | --- | --- | --- | --- | --- | --- | --- |
| **Intervention** | **None** | | **Patients** | | **GPs** | | **GPs and Patients** | |
| **Percentage of pTD patients (pre-intervention)** | 0.63 (0.44) | 1.58 (1.45) | 0.59 (0.44) | 1.25 (1.13) | 0.59 (0.42) | 2.10 (1.64) | 0.63 (0.44) | 1.59 (1.29) |
| **Pre-post difference in the percentage of pTD patients** | 0.07 (0.33) | 0.04 (0.83) | 0.13 (0.39) | 0.11 (0.99) | 0.06 (0.31) | 0.20 (1.58) | 0.09 (0.35) | -0.11 (1.72) |

**Supplementary Table 4**. Linear model on pTDs stratified by region

|  | **Estimate (95% CI)** | **p value** |
| --- | --- | --- |
| **LOMBARDY** |  |  |
| Intercept* | 0.07 (0.04; 0.11) | <0.001 |
| Intervention (Territory) |  |  |
| None (*Monza-Brianza*) | Ref. | - |
| GPs (*Bergamo*) | 0.02 (-0.01; 0.06) | 0.193 |
| Patients (*Mantova*) | 0.09 (0.05; 0.14) | <0.001 |
| GPs and patients (*Lecco*) | 0.02 (-0.02; 0.07) | 0.323 |
| GP sex |  |  |
| Female | Ref. | - |
| Male | -0.04 (-0.07; -0.01) | 0.022 |
| GP age (1-year increase) | 0.02 (-0.01; 0.04) | 0.141 |
| Number of patients (100-unit increase) | 0.02 (0.01; 0.03) | <0.001 |
| Percentage of elderly patients (1-percentage point increase) | 0.03 (0.004; 0.06) | 0.028 |
| Percentage of pre-intervention patients with duplicate drug prescriptions (1-percentage point increase) | 0.45 (0.42; 0.49) | <0.001 |
| Number of distinct drugs prescribed (10-unit increase) | -0.01 (-0.02; -0.01) | <0.001 |
| **CAMPANIA** |  |  |
| Intercept* | 0.17 (0.01; 0.32) | 0.032 |
| Intervention (Territory) |  |  |
| None (*Caserta*) | Ref. | - |
| GPs (*Napoli 1 Centro*) | 0.07 (-0.09; 0.23) | 0.415 |
| Patients (*Avellino*) | 0.18 (-0.02; 0.38) | 0.072 |
| GPs and patients (*Napoli 2 Nord*) | -0.11 (-0.27; 0.05) | 0.190 |
| GP sex |  |  |
| Female | Ref. | - |
| Male | -0.17 (-0.31; -0.03) | 0.021 |
| GP age (1-year increase) | 0.22 (0.08; 0.36) | 0.020 |
| Number of patients (100-unit increase) | 0.06 (0.03; 0.09) | <0.001 |
| Percentage of elderly patients (1-percentage point increase) | 0.02 (-0.11; 0.15) | 0.770 |
| Percentage of pre-intervention patients with duplicate drug prescriptions (1-percentage point increase) | 0.36 (0.32; 0.41) | <0.001 |
| Number of distinct drugs prescribed (10-unit increase) | -0.05 (-0.07; -0.03) | <0.001 |

GP general practitioner

* Within each region, numerical covariates were centred with respect to the mean. Therefore, the intercept represents the average outcome (pre-post difference) in the corresponding region for a female GP whose covariates were equal to the average values observed in the dataset and undergo no intervention.

**Supplementary Table 5**. Percentage of patients exposed to ERD drugs in the pre-intervention period and observed pre-post difference. Mean (SD).

| **Local Health Unit** | *Monza-Brianza*  **Lombardy** | *Caserta*  **Campania** | *Mantova*  **Lombardy** | *Avellino*  **Campania** | *Bergamo*  **Lombardy** | *Napoli 1 Centro*  **Campania** | *Lecco*  **Lombardy** | *Napoli 2 Nord*  **Campania** |
| --- | --- | --- | --- | --- | --- | --- | --- | --- |
| **Intervention** | **None** | | **Patients** | | **GPs** | | **GPs and Patients** | |
| **Percentage of ERD patients (pre-intervention)** | 26.3 (6.2) | 55.0 (11.2) | 31.5 (7.1) | 48.4 (11.9) | 29.8 (7.1) | 54.1 (12.7) | 25.9 (6.4) | 64.3 (12.3) |
| **Pre-post difference in the percentage of ERD patients** | 0.7 (4.2) | 2.1 (9.6) | 3.8 (5.6) | 4.3 (13.5) | 0.3 (4.9) | 4.3 (13.9) | -0.5 (5.0) | 11.3 (10.4) |

**Supplementary Table 6**. Linear model on ERD prescription stratified by region

|  | **Estimate (95% CI)** | **p value** |
| --- | --- | --- |
| **Lombardy** |  |  |
| Intercept* | 1.88 (1.41; 2.36) | <0.001 |
| Intervention (Territory) |  |  |
| None (*Monza-Brianza*) | Ref. | - |
| GPs (*Bergamo*) | -1.42 (-1.95; -0.89) | <0.001 |
| Patients (*Mantova*) | 1.57 (0.88; 2.25) | <0.001 |
| GPs and patients (*Lecco*) | -0.91 (-1.61; -0.22) | 0.010 |
| GP sex |  |  |
| Female | Ref. | - |
| Male | -0.87 (-1.33; -0.40) | <0.001 |
| GP age (1-year increase) | 0.08 (0.05; 0.11) | <0.001 |
| Number of patients (100-unit increase) | 0.10 (-0.04; 0.24) | 0.161 |
| Percentage of elderly patients (1-percentage point increase) | -0.01 (-0.06; 0.03) | 0.590 |
| Percentage of pre-intervention ERD patients (1-percentage point increase) | 0.34 (0.31; 0.38) | <0.001 |
| Number of distinct drugs prescribed (10-unit increase) | 0.02 (-0.10; 0.14) | 0.755 |
| **Campania** |  |  |
| Intercept* | 3.57 (2.31; 4.84) | <0.001 |
| Intervention (Territory) |  |  |
| None (*Caserta*) | Ref. | - |
| GPs (*Napoli 1 Centro*) | 2.93 (1.62; 4.24) | <0.001 |
| Patients (*Avellino*) | 4.32 (2.70; 5.93) | <0.001 |
| GPs and patients (*Napoli 2 Nord*) | 6.22 (4.88; 7.56) | <0.001 |
| GP sex |  |  |
| Female | Ref. | - |
| Male | -1.46 (-2.61; -0.30) | 0.013 |
| GP age (1-year increase) | 0.49 (0.37; 0.60) | <0.001 |
| Number of patients (100-unit increase) | 0.22 (-0.04; 0.47) | 0.094 |
| Percentage of elderly patients (1-percentage point increase) | 0.09 (-0.03; 0.21) | 0.151 |
| Percentage of pre-intervention ERD patients (1-percentage point increase) | 0.40 (0.35; 0.45) | <0.001 |
| Number of distinct drugs prescribed (10-unit increase) | -0.44 (-0.63; -0.24) | <0.001 |

GP general practitioner

* Within each region, numerical covariates were centred with respect to the mean. Therefore, the intercept represents the average outcome (pre-post difference) in the corresponding region for a female GP whose covariates were equal to the average values observed in the dataset and undergo no intervention.
